# *Lactobacillus acidophilus* Attenuates *Salmonella*-Induced Stress of Epithelial Cells by Modulating Tight-Junction Genes and Cytokine Responses

**DOI:** 10.3389/fmicb.2018.01439

**Published:** 2018-07-02

**Authors:** Alexia F. P. Lépine, Nicole de Wit, Els Oosterink, Harry Wichers, Jurriaan Mes, Paul de Vos

**Affiliations:** ^1^Section Immuno-endocrinology, Department of Pathology and Medical Biology, University Medical Center Groningen, University of Groningen, Groningen, Netherlands; ^2^Food Quality and Health Effects, Food and Biobased Research, Wageningen University & Research, Wageningen, Netherlands

**Keywords:** Caco-2, epithelial cells, gut barrier, IL-8, lactic acid bacteria, micro-array, *Salmonella*, TEER

## Abstract

**Scope:** Salmonellosis is a prevalent food-borne illness that causes diarrhea in over 130 million humans yearly and can lead to death. There is an urgent need to find alternatives to antibiotics as many salmonellae are now multidrug resistant. As such, specific beneficial bacteria and dietary fibers can be an alternative as they may prevent *Salmonella* Typhimurium (STM) infection and spreading by strengthening intestinal barrier function.

**Methods and Results:** We tested whether immune active long-chain inulin-type fructans and/or *L. acidophilus* W37, *L. brevis* W63, and *L. casei* W56 can strengthen barrier integrity of intestinal Caco-2 cells in the presence and absence of a STM. Effects of the ingredients on intestinal barrier function were first evaluated by quantifying trans-epithelial electric resistance (TEER) and regulation of gene expression by microarray. Only *L. acidophilus* had effects on TEER and modulated a group of 26 genes related to tight-junctions. Inulin-type fructans, *L. brevis* W63 and *L. casei* W56 regulated other genes, unrelated to tight-junctions. *L. acidophilus* also had unique effects on a group of six genes regulating epithelial phenotype toward follicle-associated epithelium. *L. acidophilus* W37 was therefore selected for a challenge with STM and prevented STM-induced barrier disruption and decreased secretion of IL-8.

**Conclusion:** *L. acidophilus* W37 increases TEER and can protect against STM induced disruption of gut epithelial cells integrity *in vitro*. Our results suggest that selection of specific bacterial strains for enforcing barrier function may be a promising strategy to reduce or prevent STM infections.

## Introduction

Entero-pathogens such as *Salmonella* Typhimurium (STM) actively disrupt gastrointestinal barrier function. STM does this to enhance epithelium permeability as a strategy to infect the host (Singh and Aijaz, 2015; Zhang et al., 2017). It produces virulence factors that manipulate the actin cytoskeleton of the epithelial host cells, leading to impairment of the barrier function and promoting bacterial internalization (Zhou et al., 1999; Boyle et al., 2006; Konig et al., 2016). This leads to translocation of the pathogen into the lamina propria facilitating further infection of the host and inducing salmonellosis. This is a major health care concern as salmonellosis is a prevalent food-borne illness that causes diarrheal diseases in over 130 million humans yearly^1^ and is still increasing in Europe (European Food Safety Authority, European Centre for Disease Prevention and Control, 2017). These frequent infections as well as the multiresistance to antibiotics (European Food Safety Authority, European Centre for Disease Prevention and Control, 2016) of STM makes it imperative that innovations are developed to fight STM. Limiting barrier disruption during or before STM infection to avoid invasion may be such an effective alternative therapy for antibiotics but requires careful selection of food ingredients with gut barrier protective effects.

Beneficial microbes can be actors in maintaining or stimulating barrier function, and may counteract pathogen-infection such as that of STM. Lactobacilli are particularly recognized for enhancing intestinal barrier function (Nataro and Guerrant, 2017) and to confer protective effects against multiresistant pathogens (Sambanthamoorthy et al., 2014). A lactobacilli that has anti-pathogenic effects is *L. acidophilus.* Various *L. acidophilus* strains support intestinal immune barrier function (Esvaran and Conway, 2012; Lightfoot et al., 2015; Burdick Sanchez et al., 2016) and have been shown to improve resistance to pathogens (Weiss et al., 2010), and to reduce pathogen induced traveler’s diarrhea in humans (McFarland, 2007). *In vitro*, *L. acidophilus* was shown to reduce STM adherence to epithelial cells (Candela et al., 2008). It is not known whether *L. acidophilus* also exerts protective effects on pathogen infection such as STM via modulation of tight-junctions and, consequently, via enhancement of barrier function.

Another interesting species that can modulate tight-junctions and may therefore be instrumental in the fight against STM is *L. casei.* It has been shown that *L. casei* prevented LPS-induced disruption of the gut barrier, a virulent molecule carried by STM (Yeung et al., 2013; Yang et al., 2015). *L. casei* has also been shown to modulate enterocyte inflammation related signaling as it suppressed *Clostridium difficile*-induced IL-8 production by colonic epithelial cells (Boonma et al., 2014). Another candidate, although much less studied, is *L. brevis* that can adhere to Caco-2 cells (Ramos et al., 2013). Also, fermentation products isolated from *L. brevis* suppressed mouse small intestinal permeability (Segawa et al., 2011) and may thereby reduce STM infection.

Although less extensively studied than beneficial bacteria, other food-based ingredients that can contribute to strengthening barrier function are dietary fibers (Brufau et al., 2016). For instance, inulin and FOSs have recently been shown to enhance barrier function (Wu et al., 2017) and protect against barrier dysfunction (Akbari et al., 2017). Moreover, long-chain inulin-type fructans (lcITF) reinforced barrier function via upregulated tight-junction proteins in diabetic mice (Chen et al., 2017) and in mice with acute pancreatitis (He et al., 2017). As such a protective effect of lcITF on gut barrier disruption may prevent spreading of STM.

We hypothesized that specific beneficial bacteria and dietary fibers can support barrier integrity in the event of STM infection, thereby limiting the spreading by strengthening intestinal barrier function and/or enterocyte cytokine response toward the infection. To this end, we tested lcITF and three different bacterial strains in our study on barrier (dys)function of intestinal Caco-2 cells in the presence and absence of an STM infection. Lactobacilli strains were *L. acidophilus* W37, *L. brevis* W63, and *L. casei* W56. Effects of the ingredients on intestinal barrier function were first evaluated by quantifying TEER and regulation of gene expression by microarray. The food ingredient with most effects on TEER and gene expression was then selected to evaluate if increased TEER remains during a challenge with STM and if it is accompanied by different cytokine response.

## Materials and Methods

### Ingredients and *Salmonella* Cultures in Caco-2 Experiments

LcITF with DP10-60 (Frutafit^®^ TEX!; Sensus, Cosun, Roosendaal, Netherlands) were solubilized at 0.5 mg/mL in medium and filtered (0.2 μm) to eliminate possible contaminations. The ITF was characterized by high-performance anion exchange chromatography coupled with pulsed electrochemical detection, which was performed on an ICS5000 system (Thermo Fisher Scientific, Waltham, MA, United States), equipped with a Dionex CarboPac PA-1 column (2 mm × 250 mm) in combination with a Carbopac PA-1 guard column (2 mm × 50 mm) (**Supplementary Figure S1**).

Glycerol stocks of *Lactobacillus acidophilus* W37 (LaW37), *Lactobacillus brevis* W63 (LbW63), and *Lactobacillus casei* W56 (LcW56) (Winclove, Amsterdam, Netherlands) were produced from bacteria grown anaerobically overnight in MRS medium. Upon use, glycerol stocks were washed with PBS and resuspended in DMEM (Gibco-Invitrogen, Bleiswijk, Netherlands), brought to 37°C, to reach 10^7^ CFU/mL. DMEM contained 4.5 g/L glucose, 0.58 g/L glutamine, no pyruvate, and was supplemented with 10% heat inactivated fetal calf serum (hiFCS) (Hyclone Perbio, Etten-Leur, Netherlands).

*Salmonella* Typhimurium (STM) DT12 was provided by Trouw Nutrition (Boxmeer, Netherlands). STM was grown in Brain–Heart Infusion (BHI) medium (Becton, Dickinson and Company, Le Pont de Claix, France) until stationary phase, then washed in PBS, and standardized OD value was used for further dilution to 5 × 10^6^ CFU/mL, as confirmed with CFU count on BHI plates. The ingredients used for the experiments were prepared freshly.

### Caco-2 Cell Culture and Trans-Epithelial Electric Resistance

ATCC derived Caco-2 ATCC-HTB37 cells were cultured in DMEM. Cells were used within passage numbers 30 to 40 to ensure for stability of TEER throughout the different repetitions, and 330,000 were seeded on ThinCert transwells with 33.6 mm^2^ membranes and 0.4 μm pores in 24-well suspension culture plates. Cells were grown for 21 days at 5% CO_2_ and 37°C. Wells were selected for experiments when TEER reached 330 Ohm cm^2^ with a variation <10% between wells. Apical (150 μL) and basolateral (700 μL) medium were replaced three times per week and on the day prior to the experiment.

Trans-epithelial electric resistance was measured before the stimulation with lcITF and lactobacilli using a MilliCell-ERS Ω meter (Millipore, Molsheim, France). Medium was then refreshed with or without the ingredients on the apical side. TEER was determined directly and at 1, 3, and 6 h after exposure to ingredients to determine the integrity of the confluent monolayer. Per experiment, each condition was performed in triplicate, and after 6 h incubation, the Caco-2 cells were lysed in TRIzol (Invitrogen, Life Technologies, Bleiswijk, Netherlands) and the triplicates were pooled for RNA isolation. Experiments were repeated 2–3 times, to obtain independent biological triplicates. For each time-point we calculated the delta (%) as compared to *t* = 0 for each single well, in which *t* = 0 was set to 100%. These deltas were then compared between wells exposed to compounds (lactobacilli strains and lcITF) and medium control to determine statistical significance per time point.

### *Salmonella* Typhimurium Challenge of Caco-2 Exposed to *L. acidophilus* W37

Caco-2 cells were cultured as described above but grown on ThinCert transwells of 3 μm pores to allow bacterial translocation. LaW37 was incubated on the apical side of the Caco-2 cells for 20 h, and TEER was measured at 1, 3, and 17 h during incubation with LaW37. STM strain DT12 was then added for 45 min after which the Caco-2 cells were washed in PBS, and subsequently, medium was refreshed with one containing 100 μg/mL gentamicin (DMEM^genta^). Overnight incubation led to the recovery of TEER post-challenge, which was measured after 80 min, 4 and 20 h in DMEM^genta^. Apical medium of pooled replicates was collected, centrifuged 20 min at 12,000 *g*, at 4°C, and the supernatant was stored at -80°C for IL-8 measurement.

### RNA Isolation

Total RNA was isolated as reported previously (de Wit et al., 2016). Total RNA was quantified with the Nano-drop^®^ ND-1000 Spectrophotometer (Thermo Scientific, Wilmington, DE, United States) at OD_260__nm_ and the purity was expressed with OD_260__nm_/OD_280__nm_. The quality and integrity of the RNA was confirmed on a 1% agarose gel and visualizing the 18S and 28S bands with glyxol dye. A fixed amount of 1000 μg of total RNA was used to synthesize cDNA according to manufacturer’s instructions (BioRad iScript^TM^ cDNA Synthesis kit ref). Incubation in a PCR block (MyCycles^TM^ thermal cycler, Biorad) followed the program: 5 min at 25°C; 30 min at 42°C; 5 min at 85°C. The resultant single-stranded cDNA was diluted in 40 μL of Nuclease free Milli-Q water, a pool of all samples was diluted in 20 μL to be used as standards, and they were all stored at -20°C until further use.

### Microarray

RNAs of each independent Caco-2 experiment were hybridized to Affymetrix Human Gene 1.1 ST arrays according to standard Affymetrix protocols as described previously (de Wit et al., 2016). Quality control of the datasets was performed using Bioconductor packages (Gentleman et al., 2004) integrated in an on-line pipeline (Lin et al., 2011). Array data were normalized using the RMA M-estimator method (Bolstad et al., 2003; Irizarry et al., 2003), probe sets were defined according to Dai et al. (2005). Furthermore, universal expression code analysis was performed (Piccolo et al., 2013), which is a standardized score used to describe an active/inactive state of a gene in a sample. The Bioconductor UPC package was used to assign a score to each gene in each array. Data from all microarrays can be accessed online on GEO database with accession number GSE115022. Cells were considered to have the potential to express a gene if that gene had a UPC value >0.5 in at least one array (Venkatasubramanian et al., 2017). To identify differential gene expression induced by LaW37, LcW56, LbW63, and lcITF paired-wise comparison analyses were performed (treatment versus control medium) and genes with a LIMMA raw *p*-value <0.05 were selected for further data analyses.

To gain insight into the biological role of the genes which were differently expressed between Caco-2 cells incubated with control medium or different lactobacilli strains, we performed IPA (Ingenuity System). As described previously (Elderman et al., 2017), Ingenuity uses a comprehensive expert-curated repository of biological interactions and functional annotations that follow the GO annotation principle. GO annotations are used by ingenuity in order to investigate, among others, overrepresented biological functions. The Ingenuity output includes biological functions and signaling pathways with statistical assessment of the significance of their representation based on Fisher’s exact test. Here, this test calculates the probability that genes participate in a given biological function relative to their occurrence in all other biological function annotations.

### Statistical Analysis

Trans-epithelial electric resistance values of all repetitions, expressed as delta for each time-point and normalized for time, were treated as one assay. All TEER data were normally distributed as confirmed by the Kolmogorov–Smirnov test. Statistical differences were analyzed using two-way ANOVA (analysis of variance) with LSD *post hoc* test. Data are expressed as mean ± standard error of the mean (SEM). *p*-Values <0.05 were considered to be statistically significant and *p* < 0.1 was defined as a trend. The data were analyzed with IMB SPSS Statistics 22 (IMB analytics, Armonk, NY, United States). IL-8 data were analyzed using the same procedure but with Tukey’s *post hoc* and the analysis was performed in GraphPad Prism version 7.0a (GraphPad Software, Inc., La Jolla, CA, United States).

## Results

The aim of this study was to determine whether a dietary fiber (lcITF) and different strains of lactobacilli, i.e., LaW37, LcW56 and LbW63, influence intestinal mucosal homeostasis by modulating enterocytes and epithelial cytokine responses. Effects of lcITF and lactobacilli on intestinal barrier function was tested on monolayers of Caco-2 cells, first without a challenge, and later combined with a barrier disrupting pathogen, STM DT12. Also, possible attenuating effects of the food ingredients on cytokine responses of Caco-2 cells were studied with this pathogen.

### *L. acidophilus* W37 Impacts Barrier Function via Tight-Junction Related Genes

**FIGURE 1:**
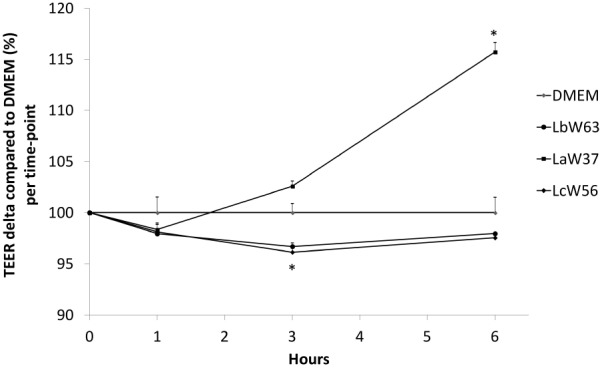
Effect of lactobacilli strains on TEER of Caco-2 cells. A 21-days differentiated Caco-2 cells were incubated with *L. acidophilus* W37, *L. brevis* W63, and *L. casei* W56 for 6 h. TEER was measured after 0, 1, 3, and 6 h incubation, values were normalized based on the time-point *t* = 0 h measurement and are expressed as delta variation compared to DMEM control for each time-point, which was set to 100%. Statistically significant difference compared to control were assessed with SPSS statistics using ANOVA followed by LSD test with ^∗^*p* < 0.05.

First, we determined *in vitro* the potential impact of lcITF and of the three *Lactobacillus* strains LaW37, LbW56, LcW63 at 1 × 10^7^ CFU/mL on the TEER of Caco-2 epithelial cells. TEER is a measure for intestinal barrier integrity. lcITF had no effect on TEER during 6 h of incubation. Also, the strains LcW63 and LbW56 had almost no effect on TEER. They both decrease TEER by 4% after 3 h incubation (LcW63 *p* = 0.03; LbW56 *p* = 0.02) but no difference was observed after 6 h (**Figure 1**). On the contrary, LaW37 enhanced TEER by 15% (*p* < 0.01) after 6 h (**Figure 1**).

To gain insight in the cause and pathways involved in this TEER enhancement by LaW37 in an unbiased way, we performed genome-wide gene expression analysis on Caco-2 cells treated with lcITF and with all three lactobacilli strains. Genes differentially expressed are all listed in **Supplementary Data Sheet S1** with additional FDR values. In addition, data from all microarrays can now be accessed online on GEO database with accession number GSE115022.

The lcITF regulated *n* = 128 (FC: -1.65 to 1.48) genes but these were not related to tight-junction regulation, nor to epithelial–immune interaction parameters. Changes of pathways in Caco-2 cells were mainly related to energy metabolism. Changes occurred at the level of genes involved in amino-acids breakdown pathways, especially the gene TAT related to tyrosine was increased, as indicated by upregulation of AUH, UBAC2, and TAT genes and downregulation of USP40. Moreover, translation initiation, TCA cycle, OxPhos and fatty-acid oxidation, were all decreased. Typical genes downregulating fatty-acid oxidation were AUH, ECI2, SDHB, SDHD, and NDUFV2. LcITF could therefore be involved in reducing energy metabolism.

Lactobacilli LcW63 and LbW56 regulated respectively, *n* = 92 (FC: -1.45 to 1.69) and *n* = 98 (FC: -1.70 to 1.38) differentially expressed genes compared to DMEM controls (*p* < 0.05). Changes were not related to tight-junction regulation, or to epithelial–immune interaction parameters. Changes occurred at the level of metabolism and coagulation with direct effects of LbW63 on two genes, PLAUR and FGA, indicating possible upregulation of growth factor signaling. Moreover, LbW63 upregulated two genes, PDIA3 and FCER1G, involved in lipid metabolism related processes. On the other hand, LcW56 differentially regulated two genes, GLUD1 and NT5C3A, involved in amino-acid metabolism; however, the direction was unclear. Besides, LcW56 also induced changes in cell proliferation with upregulation of EGF and downregulation of growth hormone signaling genes PRKD3 and GHR. Moreover, upregulation of genes PRKD3, SCNN1A, and PDIA3 by LcW56 suggested an effect on control of processes related to blood pressure while upregulation of EGF, PRKD3, and THPO suggested changes at the levels of inflammation and blood coagulation and upregulation of EGF and PRKD3 indicated increased micropinocytosis signaling.

**FIGURE 2:**
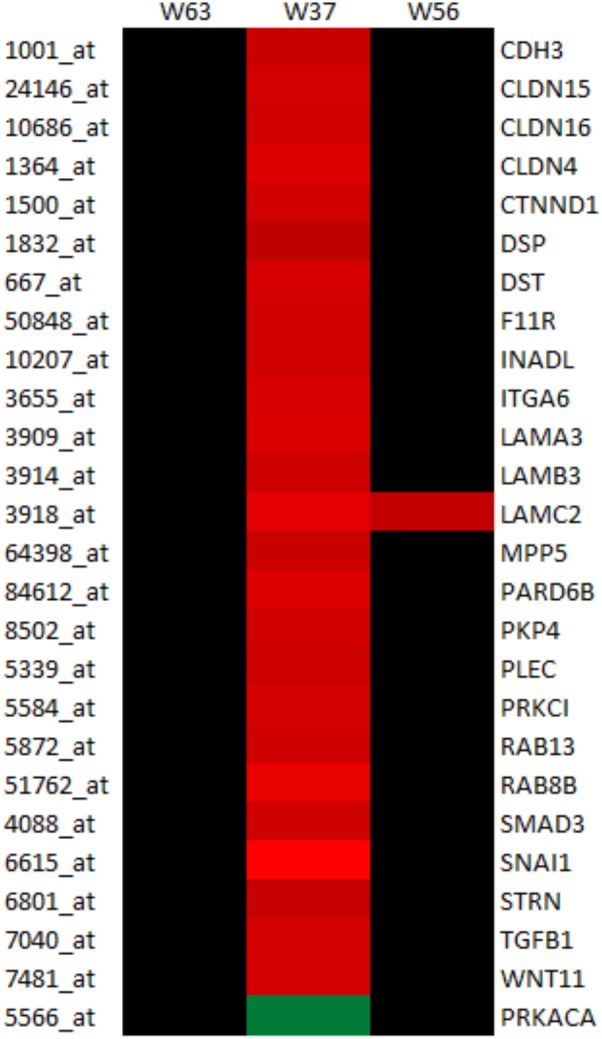
LaW37 is the only strain that enhanced tight-junction related gene expression in Caco-2 cells. Heat-map showing the relative expression of tight-junction related gene that differed significantly between Caco-2 incubated with control medium and the three lactobacilli strains. Colors indicate relative expressions normalized per gene (per row) that are statistically significantly different compared to control medium with a paired-wise LIMMA raw *t*-test and *p* < 0.05. *Dark green* is a decreased fold change, the *darkest the red* the higher the increased fold change compare to medium control, and *black* indicates no statistically significant change.

**FIGURE 3:**
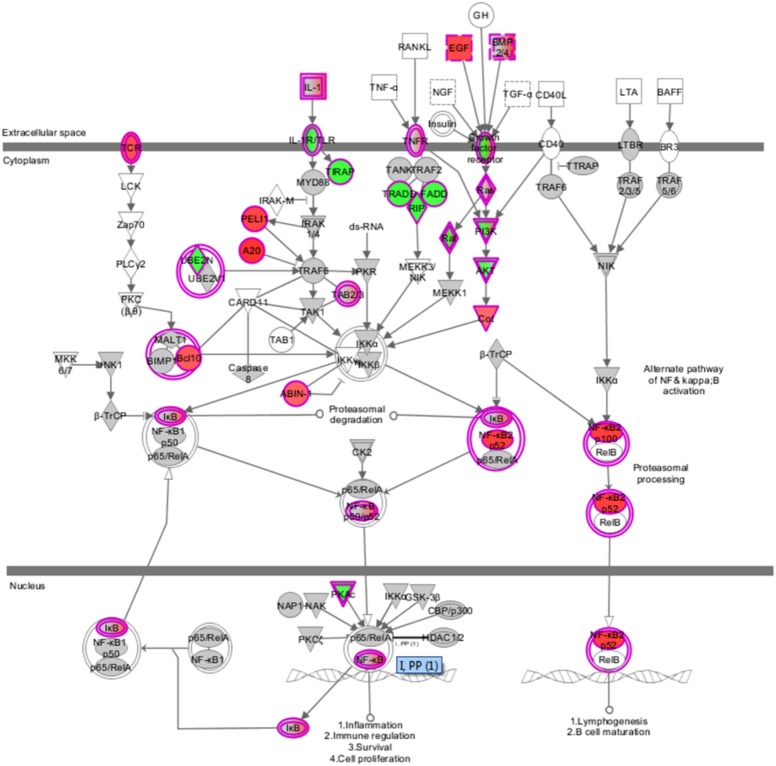
Overview of differential gene expression within NF-κB pathway induced by LaW37 in Caco-2 cells. Effects of 6 h incubation of *Lactobacillus acidophilus* W37 (LaW37) (10^7^ CFU/mL) on Caco-2 genes related NF-κB pathway signaling indicate a downregulation of this pathway. *Red* indicates upregulation and *green* indicates downregulation (*p* < 0.05).

**FIGURE 4:**
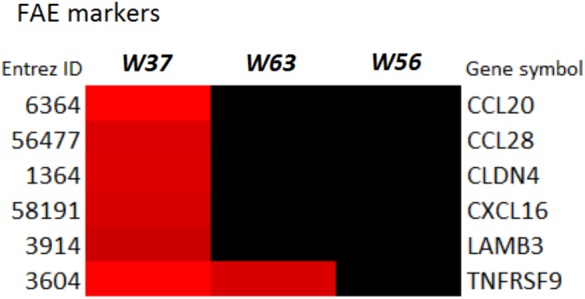
Follicle-associated epithelium (FAE) markers of array data are specifically enhanced with LaW37. Effect of lactobacilli strains (10^7^ CFU/mL) on Caco-2 genes related to FAE markers. Heatmap of differential gene expression; *red* indicates upregulation and *green* indicates downregulation (*p* < 0.05).

**FIGURE 5:**
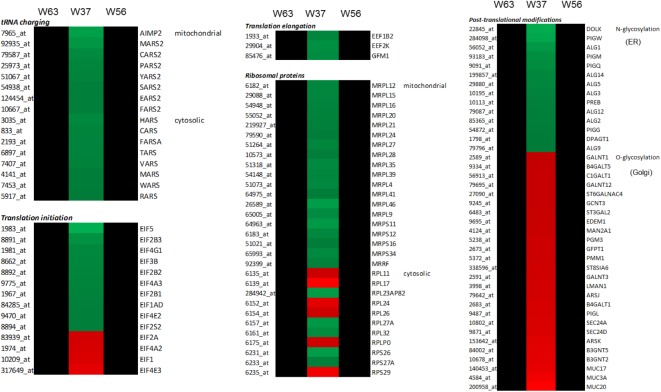
Gene expression specifically modulated by LaW37 in relation to protein metabolism in microarray data compared to LbW63 and LcW56. Effect of lactobacilli strains (10^7^ CFU/mL) on Caco-2 genes related to tRNA changes, translation initiation, translation elongation, ribosomal proteins, and post-translational modifications. Heatmap of differential gene expression; *red* indicates upregulation and *green* indicates downregulation (*p* < 0.05).

LaW37 exposure to Caco-2, however, induced differential regulation of 2743 genes (*p* < 0.05). The fold changes ranged from -2.37 to 3.62 compared to DMEM control. Most genes modulated by LaW37 were related to inflammation and bacterial stimulation of the epithelial layer. As shown on **Figure 2**, LaW37 impacted barrier function, a set of 26 genes involved in the regulation of tight-junctions were upregulated, e.g., CLDN 3, -4, and Rab13. These stimulatory effects on barrier function and possible changes in epithelium morphology seem to be highly specific for LaW37 as they were not found in Caco-2 cells incubated with other ingredients. Moreover, LaW37 seems to impact epithelial–immune interactions via TNF receptor signaling as we found a down-regulation of the canonical NF-κB pathway (**Figure 3**) as shown by downregulation of genes TNFRSF1A, TIRAP, PIK3R1, FADD, GHR, FGFR4, RIPK1, SIGIRR, UBE2N, TRADD, AKT2, ARAF, PRKACA and upregulation of NFKBIA. On the other hand, the non-canonical pathway (NF-κB2) seems to be upregulated as indicated by upregulation of the typical gene NF-κB2. A consequence of which is expected to be on IL-8 regulation as shown by differential expression of 17 genes related to IL-8 production, namely JUN, IQGAP1, PAK2, NOX1, ITGAV, ITGB2, MAP2K1, PIK3R1, PIK3CB, CCND3, FOS, GNAI2, PIK3CA, RHOT1, ROCK1, PRKCI, and GNG. Besides, LaW37 also upregulated a group of six, previously described, markers of FAE (Mabbott et al., 2013). As shown in **Figure 4**, CCL20, CCL28, CLDN4, CXCL16, LAMB3 and TNFRSF9, were substantially upregulated. Another strong effect of LaW37 was observed at the level of protein regulation (**Figure 5**). Although no pathway was clearly targeted, tRNA charging was downregulated with 16 genes related to mitochondrial and cytosolic changes; 14 genes related to translation initiation were differentially expressed; 19 genes related to mitochondrial ribosomal changes were downregulated; 11 genes related to cytosolic ribosomal changes were differentially expressed; N-glycosylation was downregulated with 14 genes concerned; O-glycosylation was strongly enhanced with 26 genes upregulated (**Figure 5**). For instance, decreased amino-acid biosynthesis was observed with downregulation of genes CARS2, YARS2, FARSA, TARS, MARS2, MARS, FARS2, PARS2, HARS, CARS, RARS, SARS2, VARS, WARS, EARS2, and downregulation of mTORC signaling was observed with differential expression of the genes RPS26, PRKAA1, RICTOR, IRS1, EIF4A2, EIF3B, PIK3R1, PIK3CB, ULK1, PIK3CA, PPP2R5A, RPS29, TSC2, PPP2CB, RHOT1, MLST8, PRKCI, EIF4A3, RND3, EIF4G1, RPS6KA4, RPS6KB2, KRAS, AKT1S1, RHOT2, MTOR, VEGFA, DDIT4, RPS27A, INSR, AKT2, PDGFC, PRKCD, NAPEPLD, RPTOR, PLD2, RHOF, RRAS, and PRKAB2. Increased cell proliferation, survival, and apoptosis was observed with upregulation of the genes IRF1, APAF1, CRABP2, CASP9, FADD, CRABP1, TNFRSF10D, TNFRSF10B, TIPARP, PARP1, CFLAR, ZC3HAV1, PARP3, TNKS2, and PA. This was combined with decreased cell cycle control as indicated by downregulation of CDK4, MCM7, CDC45, MCM2, CDK2, MCM3, ORC5, CDK5, and MCM5. Finally, upregulation of JUN, MAP2K5, MAFK, MAP2K1, PIK3CB, FOS, and JUND indicated increased oxidative stress response possibly leading to reduction of oxidative damage. Together, these results indicate that LaW37 might exert different stimulatory effects on Caco-2 including enhancement of intestinal barrier function by stimulating tight junction-related processes.

### LaW37 Protects Caco-2 Function as Measured by Decreased IL-8 Production During *Salmonella* Typhimurium Challenge

Due to the LaW37 induced enhancement of tight-junction related gene expression, we further tested if the capacity of LaW37 to increase TEER would be maintained during STM challenge and if it was connected to changes in immune function. This intra- and para-cellular invading pathogen is known to use barrier disruption as a mechanism to invade the host (Jones et al., 1994; Guttman and Finlay, 2009).

**FIGURE 6:**
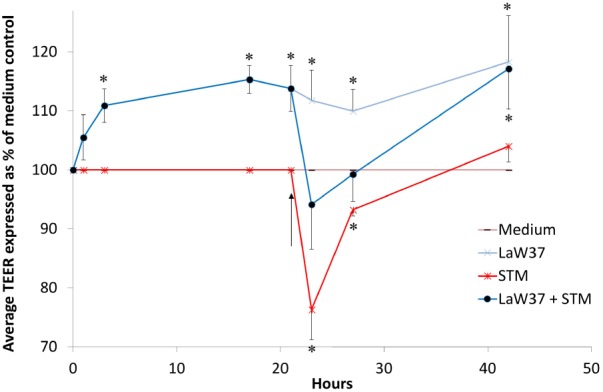
Increased TEER of Caco-2 cells with LaW37 is maintained during STM challenge. TEER values are displayed as average with SEM of triplicates Caco-2 wells grown on transwells for 21 days and are expressed as % of DMEM control with *n* = 3. STM DT12 7.5 × 10^5^ CFU/tw challenge (as indicated by the arrow) was applied for 45 min after O/N incubation (17 h) of Caco-2 cells with LaW37 10^7^ CFU/transwell. TEER was measured throughout the O/N incubation, before and after the challenge, and after O/N recovery in gentamicin medium (45 h). ANOVA with LSD *post hoc* was used to evaluate statistically significant differences (*p* < 0.05) compared to medium control at each time-point and *p* < 0.05 are indicated by asterisk (^∗^). SEM are displayed only on the positive side for the non-challenged samples and on the negative side for the samples challenged with STM.

Cells were challenged with STM which induced a drop in TEER of 24% (*p* < 0.01) compared to medium control (**Figure 6**). Although this drop was similar in terms of intensity for the cells pretreated for 17 h with LaW37, it accounted for only 3.9% (*p* < 0.05) decrease when compared to medium control. This suggests that a higher TEER acquired prior to challenge thanks to the exposure to this strain will have beneficial effect of during STM infection as compared to cells that were not exposed to LaW37.

**FIGURE 7:**
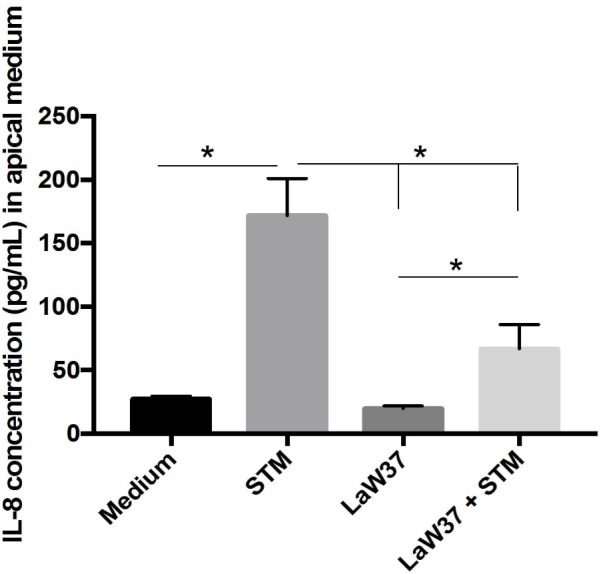
Incubation of Caco-2 with LaW37 lowers STM-associated release of IL-8 post-challenge. Average IL-8 levels released in the apical medium during an O/N recovering period after STM challenge in Caco-2 differentiated for 21 days are expressed in pg/mL. Standard deviations are displayed and statistically significant differences, analyzed with ANOVA followed by Tukey’s multiple comparisons test, are indicated by asterisk (^∗^) when *p* < 0.05.

Furthermore, we measured the concentration of IL-8 chemokine released in the medium as measure for enterocytes stress response during STM *in vitro* infection. Exposure of Caco-2 to STM for 45 min led to sixfold higher IL-8 production (*p* < 0.0001) than medium control (**Figure 7**). This was partially prevented by LaW37, as production of IL8 was only threefold higher (*p* = 0.04) compared to LaW37 control. This indicates that LaW37 not only prevents damage to barrier function but also protects from STM-induced stress response potentially via interference in the NF-κB pathway.

## Discussion

In this study, we demonstrated profound species dependent effects of *L. acidophilus* W37 (LaW37) on Caco-2 cells. We selected three strains with reported anti-pathogenic effects (Nemeth et al., 2006; Candela et al., 2008; Yeung et al., 2013; Boonma et al., 2014). Only one of the three tested bacterial strains had the capacity to enhance barrier function via upregulation of tight-junction related genes. Moreover, we show that long-chain ITF did not impact Caco-2 cells barrier integrity. The strain that enhanced barrier function, i.e., LaW37, reduced the impact of *Salmonella* Typhimurium (STM) stress by increasing TEER and decreasing IL-8 secretion. Our data therefore confirms our hypothesis that specific bacterial strains can attenuate STM induced barrier disruption.

In presence of lcITF, TEER was unaffected. Our microarray analysis shows that lcITF had no effects on tight-junctions *in vitro* in Caco-2 cells. This corroborates findings in human gut epithelial T84 cells (Vogt et al., 2014) in which only shorter chain ITF influenced barrier function. *In vivo* studies (Chen et al., 2017; He et al., 2017), however, do demonstrate effects of lcITF on barrier function. This might be explained by microbiota driven effects (Chen et al., 2017) rather than by direct effects of lcITF. Despite absence of effects on tight-junction genes we found that lcITF triggered differential regulation of 128 genes in the gut epithelial Caco-2 cells. To the best of our knowledge, it is the first time that such a direct effect of lcITF on gut epithelial cells is reported.

The strains *L. brevis* W63 and *L. casei* W56 were ineffective in enhancing TEER and even lowered TEER to some extent. Accordingly, we observed no major changes in Caco-2 gene expression. On the other hand, as expected from the observed enhancement of TEER in Caco-2 cells by LaW37, increased regulation of tight-junction gene expression was observed in our microarray analysis. LaW37 upregulated 26 genes related to barrier integrity that range from members of the claudins with upregulation of CLDN4, 15 and 16, and striatin STRN to occludins, but also proteins that are not usually checked for, such as the anchoring filament protein Laminin 5, particularly important as it was upregulated by three genes, LAMA3, LAMB3, and LAMC2. In addition, we found upregulation of tight-junction related genes that can be involved in more processes than regulation of barrier function. The PRKCI gene for instance plays a general protective role against apoptosis is also involved in NF-κB activation, cell survival, differentiation and polarity, and contributes to the regulation of microtubule dynamics in the early secretory pathway.

Interestingly, we show here that a more extensive investigation of tight-junction regulation is important as the most commonly investigated genes are claudin-1, occludin, and ZO-1 (Montalto et al., 2004; Moorthy et al., 2009; Yeung et al., 2013; Yang et al., 2015; Guo et al., 2017) and were not differently regulated by LaW37. Other studies have shown effects of various *L. acidophilus* strains, however, not LaW37, on barrier integrity and on claudin-1, occludin, and ZO-1 (Montalto et al., 2004; Moorthy et al., 2009; Yeung et al., 2013; Yang et al., 2015; Guo et al., 2017). Also, another strain of *L. acidophilus* had no effect on TEER nor on tight-junctions (Putaala et al., 2008). This suggests that within *L. acidophilus* species effects on TEER and tight-junctions is strain dependent and studying a broader set of related genes is needed to determine the mechanisms of action of bacterial strains.

In addition to effects on barrier integrity, LaW37 influenced another 2,700 genes. Many of these effects were immune related and were observed at three levels. First, LaW37 had a strong effect on TNF receptor signaling via decrease of the canonical NF-κB pathway. Secondly, LaW37 increased the non-canonical NF-κB pathway (NF-κB2), which could suggest an increase of lymphogenesis by the epithelial layer as previously observed with segmented filamentous bacteria (Atarashi et al., 2015). Downregulation of NF-κB pathways as a mean to reduce pathogenic inflammation, including *Salmonella*, was previously reported for other strains of *L. acidophilus* (Huang et al., 2015; Li et al., 2016).

Another notable observation connected to immune effects is related to changes in gene expression responsible for the formation of FAE-like cells. A group of six previously described FAE markers (Mabbott et al., 2013), namely CCL20, CCL28, CLDN4, CXCL16, LAMB3 and TNFRSF9, were substantial upregulated. This might indicate that LaW37 stimulates differentiation of enterocytes into FAE-like phenotype, a typical lining present on top of Peyer’s patches. The FAE structures contribute to antigen sampling by sensing luminal pathogens and releasing cytokine/chemokine signals that attract and activate DCs (Neutra and Kozlowski, 2006). This possible effect of LaW37 to trigger epithelial cells differentiation has never been described so far from a *Lactobacillus* bacterium.

Non-immune related functions that were differentially regulated by LaW37 are numerous and concerned decreased protein translation with downregulation of amino-acid biosynthesis and the mTORC signaling pathway. This was combined with increased cell proliferation, survival and apoptosis but also decreased cell cycle control, suggesting an increased cell turnover. Although studies investigating lactobacilli effects on cell turnover are very scarce, another lactobacilli species has been reported to induced apoptosis in gastric cancer cells by inhibiting NF-κB and mTOR-mediated signaling (Hwang et al., 2013). As we observed such effects by LaW37 on genes involved in apoptosis and regulation of both NF-κB and mTOR signaling pathways of intestinal cells, LaW37 might be instrumental in regulating abnormal behaviors in colonic cells for instance.

Pre-incubation of Caco-2 cells with LaW37 prevented STM-induced barrier disruption because of higher TEER level prior to challenge, and decreased cytokine stress responses of the epithelial cells. Release of IL8 by epithelial is known to attract neutrophils but also granulocytes to facilitate removal of STM (Eaves-Pyles et al., 2011), and is a common marker to evaluate the efficacy of lactobacilli to have therapeutic effects on epithelial cells during enteropathogenic infections (Ren et al., 2013). Our results show that IL-8 was strongly decreased by LaW37 in combination with a preserved barrier integrity. This corroborate the differential expression of 17 genes related to IL-8 production. Moreover, our array data also indicate a decreased inflammatory state with downregulated NF-κB gene expression which was specific for LaW37. Therefore, we concluded that STM-induced inflammation was reduced *in vitro* by LaW37, as previously shown for other strains (Candela et al., 2008; Yeung et al., 2013; Boonma et al., 2014; Huang et al., 2015).

## Conclusion

Screening of different lactobacilli strains on TEER in Caco-2 cells in the absence and presence of STM led to demonstration of strain dependent effects of lactobacilli on TEER and on gene expression. Only one of the three tested lactobacilli had effects on barrier integrity, and we confirmed that the selected lactobacilli, LaW37, attenuated STM induced barrier disruption as a consequence of an enhanced TEER prior to challenge. This potent effect of LaW37 makes it a relevant candidate to protect against enteropathogens such as *Salmonella*. Also, our data shows that prevention of barrier dysfunction by bacteria is strain dependent and may involve tight-junction genes that are not conventionally measured. A broader screening of relevant genes might eventually lead to identification of novel bacterial or fiber formulations that effectively prevent enteropathogen induced barrier disruption. The strategy of measuring effects of lactobacilli and dietary fibers on gut TEER in the presence of absence of STM might lead to new therapeutic strategies to reduce STM infection and may thereby contribute to reduction of use of antibiotics.

## Data Availability

All data are available upon request.

## Author Contributions

AL, NW, JM, and HW conceived and designed the experiments. AL, EO, and NW performed the experiments and analyzed the data. AL and PV wrote the paper.

## Conflict of Interest Statement

The authors declare that the research was conducted in the absence of any commercial or financial relationships that could be construed as a potential conflict of interest.

## Footnotes

1 http://www.who.int/mediacentre/factsheets/fs139/en/

## Supplementary Material

FIGURE S1 Long-chain inulin-type fructan HPAEC profile. Peaks represent fructose (F) and glucose (G) monomers, dimers, and fructans oligomers present in the formulation of lcITF. GFn and Fn chains respectively terminated by a glucose or fructose molecule with n the number of fructose moieties in the chain.

DATA SHEET S1 Differentially regulated Caco-2 genes upon exposure to long chain inulin (lcITF), and the lactobacilli strains W37, W56 and W63.

## Abbreviations

CFU: colonies forming unit
DMEM: Dulbecco’s modified eagle medium
FAE: follicle associated epithelium
FCS: fetal calf serum
FOSs: fructooligosaccharides
GO: gene ontology
IPA: ingenuity pathway analysis
ITFs: inulin type fructans
LaW37: *Lactobacillus acidophilus* W37
LbW63: *Lactobacillus brevis* W63
LcW56: *Lactobacillus casei* W56
LPS: lipopolysaccharide
PCR: polymerase chain reaction
RMA: robust multiarray average
STM: *Salmonella enterica* subspecies *enterica* serovars Typhimurium
TEER: trans-epithelial electric resistance
