# Supplementary material for: Lactobacillus acidophilus Attenuates Salmonella-Induced Stress of Epithelial Cells by Modulating Tight-Junction Genes and Cytokine Responses
Source: Front Microbiol. 2018 Jul 2;9:1439. doi: 10.3389/fmicb.2018.01439 (PMC6036613; doi:10.3389/fmicb.2018.01439)
Supplement: FIGURE S1 — Long-chain inulin-type fructan HPAEC profile. Peaks represent fructose (F) and glucose (G) monomers, dimers, and fructans oligomers present in the formulation of lcITF. GFn and Fn chains respectively terminated by a glucose or fructose molecule with n the number of fructose moieties in the chain. [file Image_1.PDF]

Supplementary Material

Lactobacillus acidophilus attenuates *Salmonella*-induced stress of epithelial cells by modulating tight-junction genes and cytokine responses

Alexia F.P. Lépine[1, 2*], Nicole de Wit[2], Els Oosterink[2], Harry Wichers[2], Jurriaan Mes[2], and Paul de Vos[1]

*** Correspondence:** A.F.P. Lépine: a.f.p.lepine@umcg.nl

Supplementary Figures

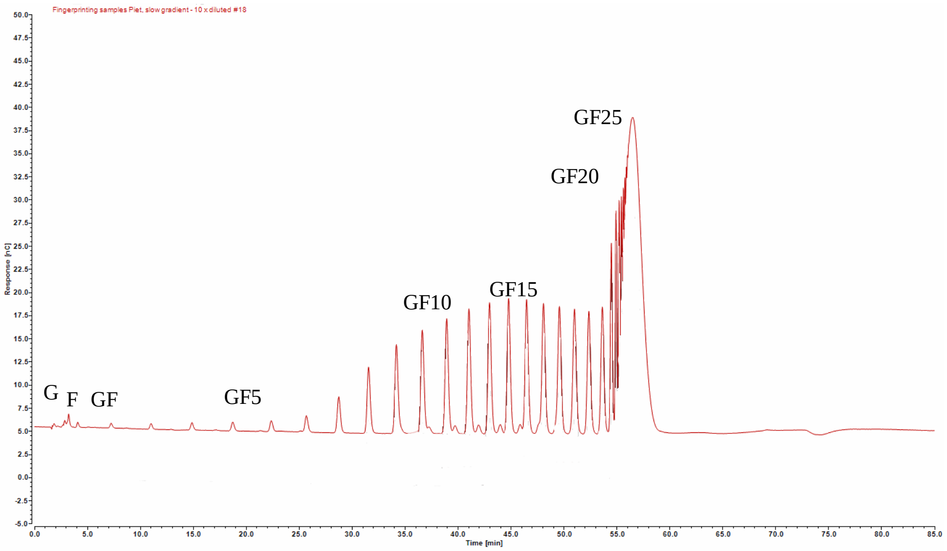

Supplementary Figure S1. Long-chain inulin-type fructan HPAEC profile. Peaks represent fructose (F) and glucose (G) monomers, dimers and fructans oligomers present in the formulation of lcITF. GFn and Fn chains respectively terminated by a glucose or fructose molecule with n the number of fructose moieties in the chain.
